# Free Triiodothyronine Connected With Metabolic Changes in Patients With Coronary Artery Disease by Interacting With Other Functional Indicators

**DOI:** 10.3389/fmolb.2021.681955

**Published:** 2021-07-30

**Authors:** Xiao-xue Tian, Shu-fen Zheng, Ju-e Liu, Yuan-yuan Wu, Lu Lin, Hong-mei Chen, Li-wen Li, Min Qin, Zi-xian Wang, Qian Zhu, Wei-hua Lai, Shilong Zhong

**Affiliations:** ^1^ School of Pharmaceutical Sciences, Southern Medical University, Guangzhou, China; ^2^ Department of Pharmacy, Guangdong Provincial People's Hospital, Guangdong Academy of Medical Sciences, Guangzhou, China; ^3^ Guangdong Provincial Key Laboratory of Coronary Heart Disease Prevention, Guangdong Cardiovascular Institute, Guangdong Provincial People’s Hospital, Guangdong Academy of Medical Sciences, Guangzhou, China; ^4^ Department of Cardiology, Guangdong Provincial People's Hospital, Guangdong Academy of Medical Sciences, Guangzhou, China

**Keywords:** free triiodothyronine, coronary artery disease, pre-brain natriuretic peptide, fibrinogen, metabolism, risk model, prognostic markers

## Abstract

This study aims to evaluate the association between free triiodothyronine (FT3) and outcomes of coronary artery disease (CAD) patients, as well as to assess the predictive power of FT3 and related functional markers from the perspective of potential mechanism. A total of 5104 CAD patients with an average follow-up of three years were enrolled into our study. Multivariate Cox regression was used to evaluate the associations between FT3, FT4 (free thyroxin), FT3/FT4 and death, MACE. We developed and validated an age, biomarker, and clinical history (ABC) model based on FT3 indicators to predict the prognosis of patients with CAD. In the multivariable Cox proportional hazards model, FT3 and FT3/FT4 were independent predictors of mortality (Adjusted HR = 0.624, 95% CI = 0.486–0.801; adjusted HR = 0.011, 95% CI = 0.002–0.07, respectively). Meanwhile, emerging markers pre-brain natriuretic peptide, fibrinogen, and albumin levels are significantly associated with low FT3 (*p* < 0.001). The new risk death score based on biomarkers can be used to well predict the outcomes of CAD patients (C index of 0.764, 95% CI = 0.731–0.797). Overall, our findings suggest that low levels of FT3 and FT3/FT4 are independent predictors of death and MACE risk in CAD patients. Besides, the prognostic model based on FT3 provides a useful tool for the death risk stratification of CAD patients.

## Introduction

Coronary artery disease (CAD), also known as coronary atherosclerotic heart disease (CHD), is an inflammatory atherosclerotic disease that manifests as stable angina, unstable angina, myocardial infarction, and sudden cardiac death (álvarez-álvarez et al., 2017.; Pothineni et al., 2017; Musunuru and Kathiresan, 2019). Despite the prominent enrichment of treatments, patients with CAD have poor prognoses and high mortality (Li et al., 2015; Elmariah et al., 2018). The prognosis of CAD is affected by many factors, and the prognostic value of traditional risk factors for CAD is limited. A complex relationship exists between thyroid hormone (THs) levels and outcomes of CAD. THs play a central role in many cellular processes, including differentiation, growth, metabolism, and physiology (Boelaert; Gorman, 2006). Changes in TH concentrations in plasma, especially low triiodothyronine (T3) levels, represent hormonal imbalance, which is usual among patients suffering from an acute coronary event (Iervasi, 2008; Xu, 2012; Hao, 2014).

T3, the biologically active form of THs, is derived mainly from the peripheral transformation of precursor thyroxine (T4). Variations in the concentrations of T3 and T4 in plasma may exert a wide range of functions in several mechanisms, including heart dysfunction (Iervasi et al., 2003; Yazıcı et al., 2017). The levels of free triiodothyronine (FT3) and free thyroxin (FT4) are important indicators of the metabolic status of THs and have gained increasing attention as markers of many acute diseases (Iervasi et al., 2003; Zhang et al., 2016). The value of FT3 as an independent risk factor for the prognosis of patients with CAD remains controversial (Coceani et al., 2010; Wang et al., 2013; Lamprou et al., 2017; Wang et al., 2017). In addition, guidelines recommended for routine screening of low T3 syndrome in patients with CAD have not been pushed (Levine et al., 2016). Low T3 syndrome, known as nonthyroidal illness syndrome and euthyroid sick syndrome, is characterized by low serum levels of total T3 and FT3 with normal levels of thyroid stimulating hormone (TSH) and FT4; this condition is deemed as a strong prognostic determinant of chronic and systolic heart failure (HF) (Iervasi et al., 2003; Cooper and Biondi, 2012; Zhang et al., 2012; Jabbar et al., 2015; Yazıcı et al., 2017).

To the best of our knowledge, precise identification and discriminatory risk evaluation are important prerequisites to targeted treatment and prevention in high risk of all-cause death and major adverse cardiovascular (MACE) for CAD. Identifying novel and overlooked biomarkers, which not only guide the diagnosis and prognosis of patients with CAD but also detect new molecular mechanisms to elucidate the pathological progress of CAD, is important due to the limited predictive power of few predictors based on genetic factors and traditional clinical risk biomarkers available for risk stratification in patients with CAD. Hyperthyroidism is linked to an increased risk of thrombus (Kim et al., 2017). Data from epidemiological studies indicate that patients with low thyroid hormone levels are at higher risk of heart failure, and the prognosis of heart failure is also worse (Vale et al., 2019). A study suggested that a rise in thyroxine level is associated with the increase of FVIII, FIX, VWF and fibrinogen levels (Debeij et al., 2010). It has been reported that there is a positive correlation between FT3 level and cardiac ejection fraction, and a significant negative correlation with NT-proBNP. Although the imbalance of FT3 and FT4 levels is the most common manifestation of thyroid dysfunctions in patients with CAD, previous data on the prevalence of low FT3 and high FT4 levels in CAD patients are insufficient, and their prognostic roles are unclear. Thus, we evaluated the association of FT3 with other functional parameters and outcomes in 5104 CAD patients and assessed the predictive power of FT3 combined with other markers. The levels of thyroid hormone and related biomarkers are predictive of death in CAD, likely expressing different and synergistic pathogenic pathways, and their combined assay significantly improves the ability of risk stratification.

## Methods

### Study Populations

During the prospective study period, 5,104 Chinese CAD participants from Guangdong Provincial People’s Hospital, who underwent coronary angiography, were included. Patients were consecutively enrolled between January 2010 and December 2013 and followed up for all-cause death and MACE up to five years. The exclusion criteria for patients in single-center cohort study included the following: 1) age <18 years or >80 years, 2) renal insufficiency (defined as serum creatinine concentration > two times the upper limit of normal [230 μmol/L], history of renal transplantation or dialysis), 3) hepatic insufficiency (defined as serum transaminase concentration > two times the upper limit of normal [80 U/L], or a diagnosis of cirrhosis), 4) being pregnant or lactating, 5) advanced cancer or hemodialysis, 6) history of thyroid problems and use of antithyroid drugs or thyroid hormone medication, and 7) incomplete information about cardiovascular events during follow-up.

The primary endpoint of interest was all-cause death, followed by MACE. MACE is the occurrence of cardiac death, nonfatal myocardial infarctions, coronary revascularization, and cerebral infarction. All participants were followed up prospectively for the study endpoints by inpatient and outpatient hospital visits and telephone contacts with the patients or their families. At each follow-up assessment (every 6 months), the participants were questioned about new adverse cardiovascular events. From August 2010 to August 2018, all patients were followed up for the primary endpoint (all-cause death) and secondary endpoint (MACE), with a mean follow-up time of 3 years. Baseline information, including demographics, medical history, biochemical measurements, and medication, were obtained from the hospital information database. This study was approved by the Medical Ethical Review Committee of Guangdong Provincial People’s Hospital and conducted according to the Declaration of Helsinki. Informed consent was obtained from all individual participants included in the study.

### Study Design

Eligible participants were evaluated for demographic factors (e.g., age and gender), clinical characteristics (e.g., The Synergy between PCI with TAXUS and Cardiac Surgery score, left ventricular ejection fraction [LVEF]), CAD risk factors (e.g., diabetes mellitus, smoking, hypertension, body mass index, and dyslipidemia), and current medication use. Baseline characteristics, including demographics, medical history, biochemical measurements, and medication, were obtained from the hospital information database. Follow-up data on mortality (time and cause of death) were used in the analysis as the primary outcome of interest. In some cases, these outcome data were unavailable. The serum concentrations of T3, FT3, T4, FT4, and of TSH have been determined and recorded. Normal range was as follows: T3, from 70 ng/dl to 170 ng/dl; FT3, from 3.28 to 6.47 pmol/L; T4, from 4.5 μg/dl to 12.0 μg/dl; FT4, from 7.64 to 16.03 pmol/L; TSH, from 0.49 to 4.91 μIU/ml, pro-brain-type natriuretic peptide (proBNP), from 0 to 125 pg/ml.

### Statistical Analysis

Continuous variables were expressed as mean ± SD, and categorical variables are described using number and percentage. Student’s t-test was used to compare continuously normal variables, the Wilcoxon rank-sum test was used for continuously abnormal variables, and the χ^2^-test was used for categorical variables. Cox proportional hazards analysis was performed to investigate the associations of clinical parameters, levels of FT3, FT4 or FT3/FT4 ratio with outcomes. In the multivariable analysis. We implemented three models to evaluate the impact of potential confounders. Model 1 included age, sex. Model 2 further adjusted for a history of diabetes, heart failure, aspartate aminotransferase, apolipoprotein a, creatine, creatine kinase MB, high-density lipoprotein cholesterol, glucose, lipoprotein (a), medication history of calcium channel blockers and proton pump inhibitors. Model 3 incorporated the components of model 2, heart rate, systolic blood pressure and diastolic blood pressure. Variables with *p* < 0.05 in the univariate Cox regression analysis were included in the multivariate Cox proportional hazard regression models by an improved backward stepwise procedure to select covariates based on the Akaike information criterion (AIC). The Spearman rank correlation coefficient (Spearman ρ) was used to determine the associations among FT3, FT4, FT3/FT4, and other risk factors. In addition, the Harrell–Lee C-index was used to quantify the additional predictive value of the new model over the traditional model containing common clinical variables. The area under the curve (AUC) of time-dependent receiver-operating characteristic (ROC) analysis was used to quantify the predictive performance. In addition, each selected biomarker was assigned a corresponding score based on its value on the nomogram. Thus, the total score of death and MACE risk was calculated by nomogram, and the cutoff points of the total score detected by x-tile analysis could be used to divide CAD patients into different categories.

The final model was represented as a nomogram. Calibration curves (2000 bootstrap resamples) were generated to verify the accuracy of the nomogram. Decision curve analyses were performed to assess the clinical utility of the nomogram. Finally, we used X-tile software (Version 3.6.2, calculated by the “rms package” of R software) to calculate the optimal cut-off points for linear prediction and established a prognostic risk stratification. Kaplan-Meier method was used to estimate the survival probabilities and obtain survival curves, which are used to illustrate the cumulative incidence of clinical endpoints based on the cut-off values, and the log-rank (Mantel–Cox) test was adopted to compare survival curves. A two-tailed *p* < 0.05 was statistically significant. All tests were performed using SAS (version 9.4) and R (version 3.6.2, http://www.R-project.org/).

## Results

### Baseline Characteristics

**FIGURE 1:**
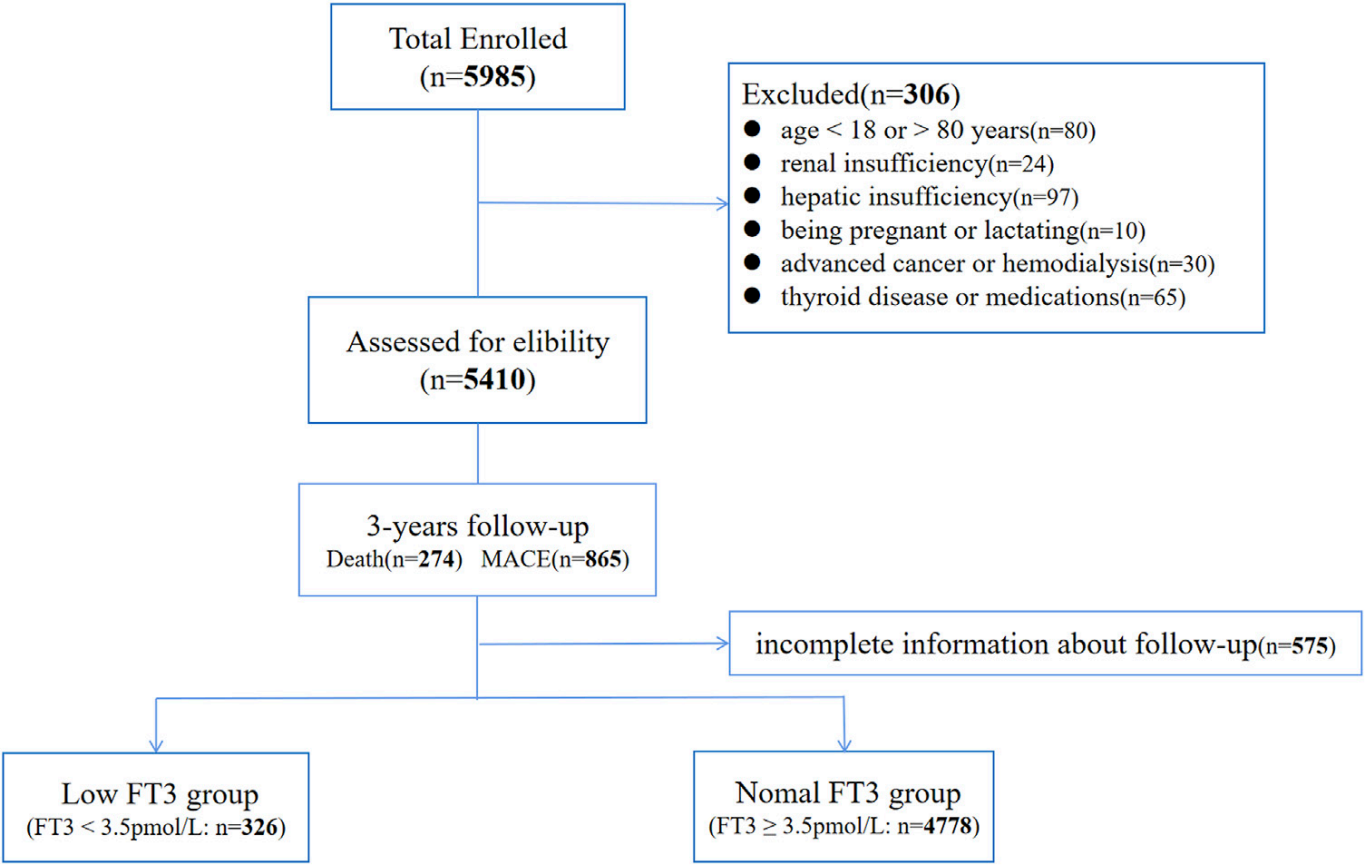
Study design (Flowchart of the study population).

A total of 5104 CAD patients were investigated, including 3,886 males (76.3%) and 1,218 females (23.7%), with an average age of 64.2 years at baseline, and a flowchart of the study population is shown in Figure 1. Table 1 provides the baseline characteristics of the participants. During an average of 3 years of follow-up, 274 (5.61%) died and 865 (16.9%) had MACE events. The mean ± standard deviations of FT3, FT4, and FT3/FT4 were 4.4 ± 0.93 pmol/L, 11.73 ± 2.86 pmol/L, and 0.39 ± 0.1 pmol/L, respectively. FT3 < 3.5 pmol/L and FT4 > 17.8 pmol/L are divided into low FT3 group (*N* = 326) and high FT4 group (*N* = 105). In addition, patients with FT3/FT4 <0.33 were classified as the low FT3/FT4 group (*N* = 1,194) in accordance with the lower quartile lower limit. The proportion of patients with HF was higher in the low FT3 group (14.11 vs 6.23%, *p* < 0.0001), and no significant difference was found in the proportion of patients with smoking history. The common clinical indicators in the low FT3 group were higher than those in the normal FT3 group. Myocardial damage indexes of proBNP, hydroxybutyrate dehydrogenase (HBDH), cardiac troponin (cTnl), high-sensitivity C-reactive protein (hs-CRP), and high-sensitivity cardiac troponin (hs-cTnl) in the low FT3 group were higher than those in the normal FT3 group (all *p* < 0.001). However, the level of albumin (ALB), triglyceride (TRIG), total protein (TP), hemoglobin (HGB), and lymphocyte in the low FT3 group was lower than that in the normal FT3 group (Supplementary Table S1).

**TABLE 1:** Baseline characteristics.

Characteristics	Value N (%) or mean ± SD			
	Total (N = 5,104)	Low FT3 (N = 326)	Normal FT3(N = 4,778)	*p* Value
Demographic data				
Age	64.17 ± 10.61	69.79 ± 9.35	63.79 ± 10.57	<0.0001
Sex (male)	3,886 (76.3%)	224 (69.14%)	3,632 (76.84%)	0.0017
BMI, kg/m2	24.68 ± 4.99	23.84 ± 4.59	24.73 ± 5.02	0.0005
Smoke	977 (21.72%)	56 (22.13%)	912 (21.69%)	0.8695
Comorbidities				
Arrhythmia	481 (9.43%)	42 (12.88%)	435 (9.19%)	0.0272
Diabetes	1,425 (27.92%)	110 (33.74%)	1,300 (27.46%)	0.052
Heart failure	346 (6.78%)	46 (14.11%)	295 (6.23%)	<0.0001
Hypertension	3,053 (59.82%)	213 (65.34%)	2,811 (59.35%)	0.0356
Hyperlipidemia	623 (12.21%)	32 (9.82%)	589 (12.44%)	0.173
Baseline biochemical measurements				
ALT, U/L	31.31 ± 32.69	41.09 ± 66.79	30.67 ± 28.89	0.1013
AST, U/L	35.64 ± 55.71	59.37 ± 106.21	34.07 ± 50.29	<0.0001
CK, U/L	173.38 ± 435.84	296.58 ± 620.98	165.63 ± 420.73	0.0497
eGFR, ml/min/1.73 m^2^	100.37 ± 365.33	66.62 ± 29.07	102.94 ± 379.86	<0.0001
CKMB, U/L	10.52 ± 18.4	13 ± 24.26	10.37 ± 18	0.5443
CHOL, mmol/L	4.48 ± 1.21	4.51 ± 1.51	4.48 ± 1.19	0.5021
LDLC, mmol/L	2.76 ± 0.97	2.73 ± 1.14	2.77 ± 0.96	0.0769
HDLC, mmol/L	0.98 ± 0.24	0.96 ± 0.27	0.99 ± 0.24	0.0566
TRIG, mmol/L	1.68 ± 1.31	1.61 ± 1.52	1.69 ± 1.29	0.0007
GLUC, mmol/L	6.79 ± 2.99	7.43 ± 3.22	6.74 ± 2.97	<0.0001
Lpa, mg/L	290.4 ± 325.83	353.84 ± 377.45	286.53 ± 321.31	0.0004
APOA, g/L	1.09 ± 0.26	1 ± 0.26	1.09 ± 0.26	<0.0001
TSH,mu/L	1.93 ± 3.47	3.72 ± 10.68	1.82 ± 2.22	0.5547
FT4,pmol/L	11.72 ± 2.86	11.85 ± 3.29	11.62 ± 2.22	0.0856
Medication				
β-blockers	3,746 (76.39%)	258 (79.63%)	3,457 (76.16%)	0.155
ACEIs	2,612 (53.32%)	195 (60.19%)	2,394 (52.8%)	0.0103
CCBs	1,458 (29.76%)	111 (34.26%)	1,334 (29.42%)	0.0669
PPIs	2,356 (48.09%)	206 (63.58%)	2,136 (47.11%)	<0.0001

SD = standard deviation; BMI = body mass index; ALT = alanine aminotransferase; AST = aspartate aminotransferase; CK = creatine kinase; eGFR = estimated glomerular filtration rate; CKMB = creatine kinase MB; CHOL = cholesterol; LDLC = low-density lipoprotein cholesterol; HDLC = high-density lipoprotein cholesterol; TRIG = triglyceride; GLUC = glucose; Lpa = lipoprotein (a); APOA = apolipoprotein a; ACEIs = angiotensin converting enzyme inhibitors; CCBs = calcium channel blockers; PPIs = proton pump inhibitors.

### Associations Between Thyroid Hormones and Other Functional Parameters

A significant correlation exists between FT3/FT4 and ALB (*r* = 0.381, *p* < 0.001) and followed by proBNP (*r* = −0.285, *p* < 0.001). The results showed that ALB (*r* = 0.348), TP (*r* = 0.268), HCT (*r* = 0.279), and HGB (*r* = 0.276) were positively correlated with FT3 concentration (*p* < 0.001). Meanwhile, proBNP (*r* = −0.194), HBDH (*r* = −0.156), and DBIL (*r* = −0.161), and myocardial injury indicators Troponin I, high-sensitivity troponin T were negatively correlated with FT3 concentration (*p* < 0.001) (Table 2), Similar patterns of correlation strength were also found in FT3/FT4 ratio. In addition, statistically significant associations were also found between coagulation markers and thyroid hormone levels.

**TABLE 2:** Cox proportional hazards analysis for Death.

Characteristics	Univariate analysis		Multivariate analysis	
	HR (95%CI)	*p* value	HR (95%CI)	*p* value
Demographic data				
Age	1.055 (1.041–1.069)	3.33E-15	1.035 (1.02–1.051)	5.34E-06
Sex	1.098 (0.823–1.465)	0.5248		
Smoke	1.786 (1.209–2.639)	0.0036		
BMI	0.914 (0.864–0.966)	0.0015		
Comorbidities				
Arrhythmia	2.11 (1.555–2.862)	1.63E-06		
Diabetes	1.378 (1.155–1.643)	0.0004		
Heart failure	4.048 (3.071–5.334)	3.11E-23	2.484 (1.808–3.412)	1.94E-08
Hypertension	1.461 (1.133–1.882)	0.0034		
Hyperlipidemia	0.612 (0.388–0.965)	0.0345		
Medication				
β-blockers	0.965 (0.707–1.318)	0.8248		
ACEIs	1.088 (0.85–1.393)	0.5019		
CCBs	1.552 (1.212–1.985)	0.0005		
PPIs	1.291 (1.013–1.646)	0.0392		
Biochemical measurements				
eGFR	0.979 (0.974–0.984)	4.34E–15		
ALT	1.001 (0.999–1.004)	0.2539		
AST	1.002 (1–1.003)	0.0061		
APOA	0.366 (0.218–0.612)	0.0001	0.328 (0.18–0.597)	0.00026
CHOL	0.875 (0.786–0.973)	0.014		
CK	1 (1–1)	0.0385		
CKMB	1.004 (1–1.009)	0.0527		
GLUC	1.067 (1.039–1.096)	2.35E–06	1.068 (1.03–1.108)	0.0003
HDLC	0.484 (0.294–0.797)	0.0043		
LDLC	0.864 (0.758–0.985)	0.0283		
LPa	1.001 (1–1.001)	0.0004	1 (1–1)	0.018
TRIG	0.914 (0.813–1.028)	0.1343		
FT3	0.411 (0.336–0.503)	7.69E–18	0.577 (0.454–0.734)	7.17E-06
FT4	1.051 (1.032–1.069)	3.49E–08		
FT3/FT4	4.12E-04 (9.05E-05-0.00188)	7.45E–24	0.01 (0.001–0.064)	7.37E-07
TSH	0.998 (0.966–1.032)	0.9268		

### Clinical Outcomes

**FIGURE 2:**
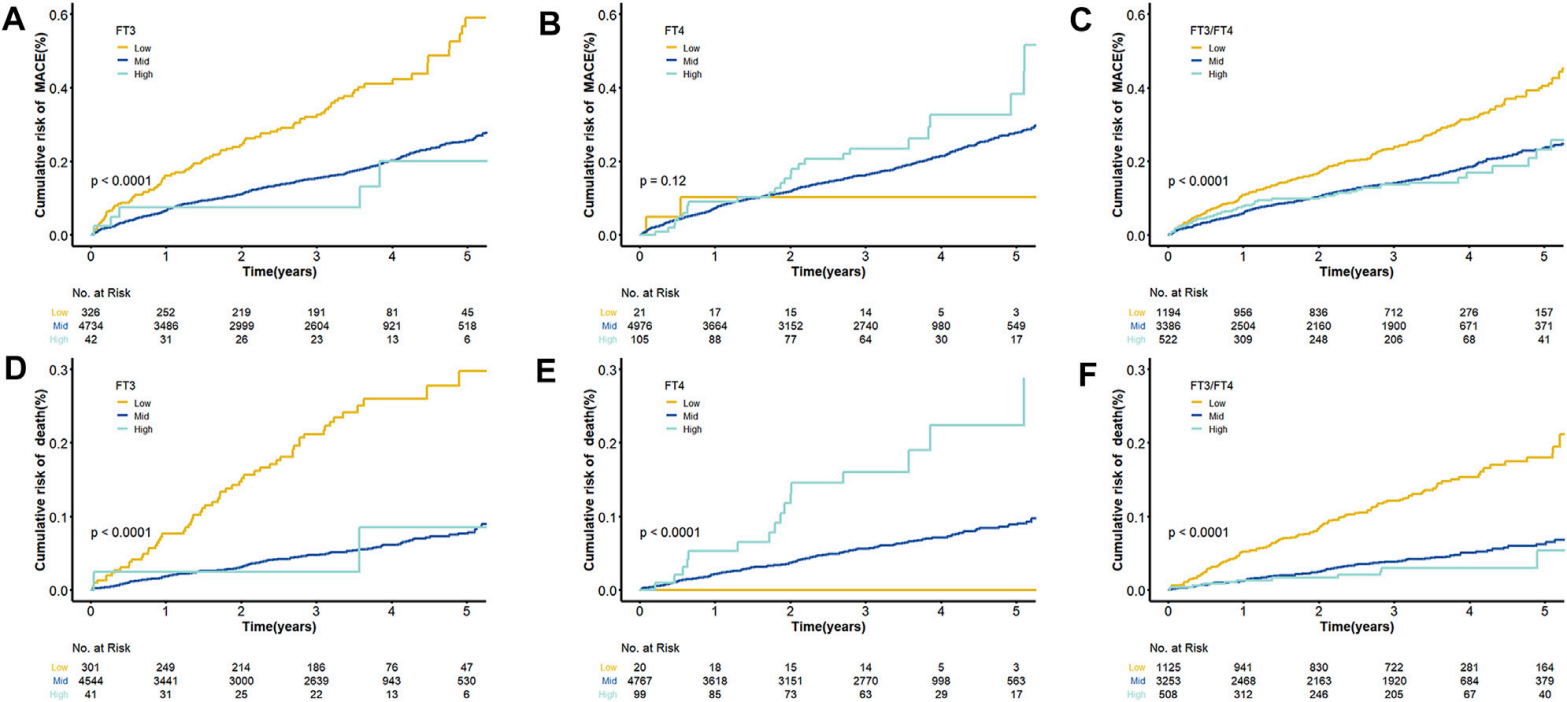
Cumulative Kaplan–Meier Curves for FT3, FT4 and the ratio of FT3 and FT4. **(A)** Cumulative Kaplan–Meier Curves for MACE according to FT3 quartiles; **(B)** Cumulative Kaplan–Meier Curves for MACE according to FT4 quartiles; **(C)** Cumulative Kaplan–Meier Curves for MACE according to FT3/FT4 quartiles; **(D)** Cumulative Kaplan–Meier Curves for death according to FT3 quartiles; **(E)** Cumulative Kaplan–Meier Curves for death according to FT4 quartiles; **(F)** Cumulative Kaplan–Meier Curves for death according to FT3/FT4 quartiles.

After an average of three years of follow-up, 116 (35.58%) of the 326 patients had MACE events and 63 (20.93%) died in the low FT3 group. Supplementary Tables S2–S3 provides univariable Cox modeling results for clinical indices and biomarkers in relation to mortality and MACE. As shown, age conferred increased risk for death (HR = 1.055, 95% CI = 1.041–1.069, and *p* = 3.33E-15). Biomarker data demonstrated a strong association between elevated FIB and risk for death (HR = 1.33, 95% CI = 1.23–1.43, and *p* = 8.06E-13). Low ALB levels may prompt increased death MACE risks (HR = 0.859, 95% CI = 0.837–0.881, and *p* = 3.98E-32; HR = 0.933, 95% CI = 0.918–0.947, and *p* = 6.98E-18, respectively). In the multivariable Cox regression analyses, FT3, FT4 and FT3/FT4 were all independent predictors of mortality after controlling for age and other clinical markers (*p* < 0.05) (Table 3). Notably, multivariate regression yielded similar results regarding relation between FT3, FT3/FT4 and MCACE risk, while after adjustment by clinical variables, no significant correlation was observed between FT4 and MACE risk (Adjusted HR = 1.008, 95% CI = 0.985–1.032). The cumulative survival curves of MACE and death in patients with different FT3, FT4, and FT3/FT4 levels are shown in Figure 2. Results demonstrate consistently the prognositic difference in different FT3, FT4 or FT3/FT4 ratio groups.

**TABLE 3:** Cox proportional hazards analysis for MACE.

Characteristics	Univariate analysis		Multivariate analysis	
	HR (95%CI)	*p* value	HR (95%CI)	*p* value
Demographic data				
Age	1.014 (1.007–1.021)	0.0002	1.008 (1–1.016)	0.032
Sex	1.218 (1.03–1.441)	0.0212	1.281 (1.039–1.581)	0.02
Smoke	1.092 (0.881–1.353)	0.4218		
BMI	0.982 (0.96–1.004)	0.1023		
Comorbidities				
Arrhythmia	1.296 (1.056–1.589)	0.0129		
Diabetes	1.345 (1.212–1.492)	2.54E-08	1.44 (1.218–1.704)	1.94E-05
Heart failure	1.934 (1.584–2.36)	8.60E-11	1.795 (1.463–2.201)	1.90E-08
Hypertension	1.274 (1.108–1.465)	0.0007		
Hyperlipidemia	1.051 (0.855–1.293)	0.637		
Medication				
β-blockers	0.907 (0.768–1.072)	0.2519		
ACEIs	1.025 (0.893–1.176)	0.73		
CCBs	1.432 (1.245–1.647)	5.27E-07	1.366 (1.152–1.62)	0.0003
PPIs	1.207 (1.053–1.384)	0.0068		
Biochemical measurements				
eGFR	0.998 (0.997–1)	0.0487		
ALT	1.001 (1–1.003)	0.1452		
AST	1.001 (1.001–1.002)	5.15E-05		
APOA	0.532 (0.404–0.699)	6.09E-06		
CHOL	1.032 (0.979–1.089)	0.2405		
CK	1 (1–1)	6.00E-04		
CKMB	1.004 (1.002–1.007)	4.00E-04		
GLUC	1.047 (1.028–1.066)	1.01E-06		
HDLC	0.514 (0.388–0.68)	3.17E-06		
LDLC	1.055 (0.987–1.128)	0.1144		
LPa	1 (1–1.001)	5.30E-06	1 (1–1)	0.0003
TRIG	0.991 (0.941–1.042)	0.7163		
FT3	0.79 (0.71–0.879)	1.42E-05	0.831 (0.735–0.939)	0.003
FT4	1.027 (1.011–1.044)	0.0009		
FT3/FT4	0.104 (0.0465–0.233)	3.54E-08	0.312 (0.121–0.8)	0.015
TSH	0.985 (0.959–1.011)	0.254		

### Development and Validation of the Predictive Model Based on FT3 and FT4

**FIGURE 3:**
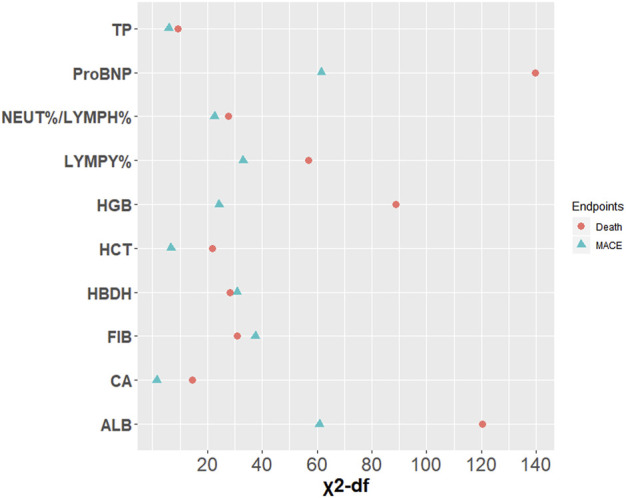
Relative importance of each variable in the full model. Measured by partial Wald χ^2^ minus the predictor degrees of freedom.

After univariate Cox regression analysis of 15 indicators significantly related to FT3 and FT4 (Supplementary Table S3), the indicators with *p* < 0.05 were screened out as the candidate variables for subsequent model constructions. A model including all candidate predictors were fitted. Then, the importance of each predictor variable in the model was measured by partial chi-square statistics minus degrees of freedom. Finally, the top three relevant candidate indicators were obtained. In the fitted predictive death model, proBNP (*X* ^*2*^ *-*df = 139.77), ALB (*X* ^*2*^ *-*df = 120.33), and HGB (*X* ^*2*^ *-*df = 88.67) were significantly correlated with death. However, proBNP (*X* ^*2*^ *-*df = 61.56) and ALB (*X* ^*2*^ *-*df = 60.97) were strongly independent predictors of MACE, and FIB (*X* ^*2*^ *-*df = 37.56) had greater prognostic value than other candidate variables involved in the outcome of MACE (Figure 3).

**FIGURE 4:**
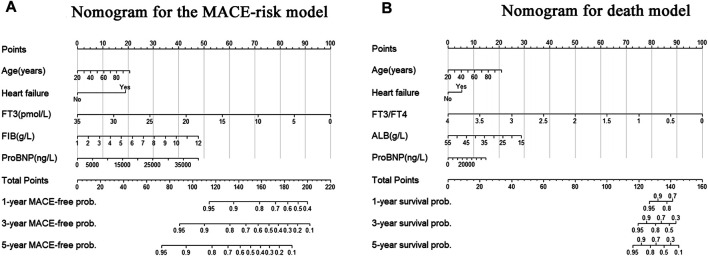
Nomogram for the new biomarker-based risk score. **(A)** Nomogram for the final biomarker-based ABC-MACE risk score; **(B)** Nomogram for the final biomarker-based ABC-death risk score. Note: The scores of each variable were added to obtain the total score, and a vertical line was drawn on the total score to obtain the corresponding probability of outcomes.

Our aim is to develop and validate a new predictive model based on FT3, including age, biomarker, and clinical history (ABC). HF, which was significantly correlated with endpoint events after multivariate Cox correction based on AIC stepwise regression, was selected as the clinical variable. The final predictive model established by age and candidate markers was represented via a nomogram (Figure 4).

**FIGURE 5:**
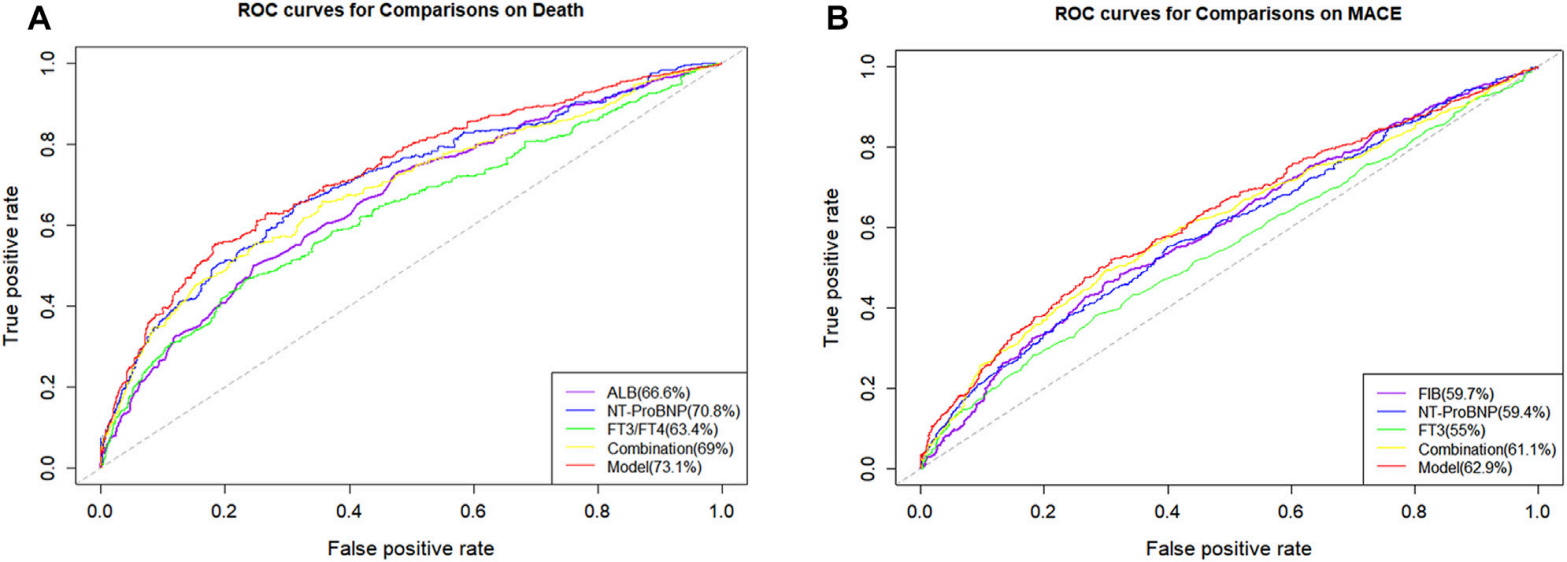
Receiver operating characteristics curves (ROC) of different parameters and models. **(A)** ROC curves of different parameters and models for predicting all-cause mortality; **(B)** ROC curves of different parameters and models for predicting MACE.

On the one hand, the final MACE predictive models including age, HF, FT3, FIB, and proBNP showed a C index of 0.621 (95% CI = 0.596–0.646), which was significantly higher than the traditional model consisting of common clinical variables (C index of 0.586, 95% CI = 0.554–0.618) (Table 4). On the other hand, the death predictive models of the single AUC of ALB, proBNP, and FT3/FT4 obtained 0.666, 0.708, and 0.634, respectively. The MACE predictive models of the single AUC of FIB, proBNP, and FT3 obtained 0.597, 0.594, and 0.55, respectively (Figure 5).

**TABLE 4:** C indices of models.

Model	C Index (95%CI)	
	Death	MACE
Model 1	0.693 (0.633–0.752)	0.586 (0.554–0.618)
Model 2	0.751 (0.715–0.787)	0.621 (0.596–0.646)
Model 3	0.764 (0.731–0.797)	0.614 (0.590–0.638)
Model 4	0.758 (0.724–0.793)	0.619 (0.596–0.643)
Model 1 of Death: Traditional model (age + sex + HyperT + DM + CHOL + HDLC + BMI + smoking + TRIG)		
Model 2 of Death:age + HF + FT3/FT4+HGB + ProBNP		
Model 3 of Death:age + HF + FT3/FT4+ALB + ProBNP		
Model 4 of Death:age + HF + FT3/FT4+ALB + HGB		
Model 1 of MACE: Traditional model (age + sex + HyperT + DM + CHOL + HDLC + BMI + smoking + TRIG)		
Model 2 of MACE:age + HF + FT3+FIB + ProBNP		
Model 3 of MACE:age + HF + FT3+ALB + ProBNP		
Model 4 of MACE:age + HF + FT3+ALB + FIB		

**FIGURE 6:**
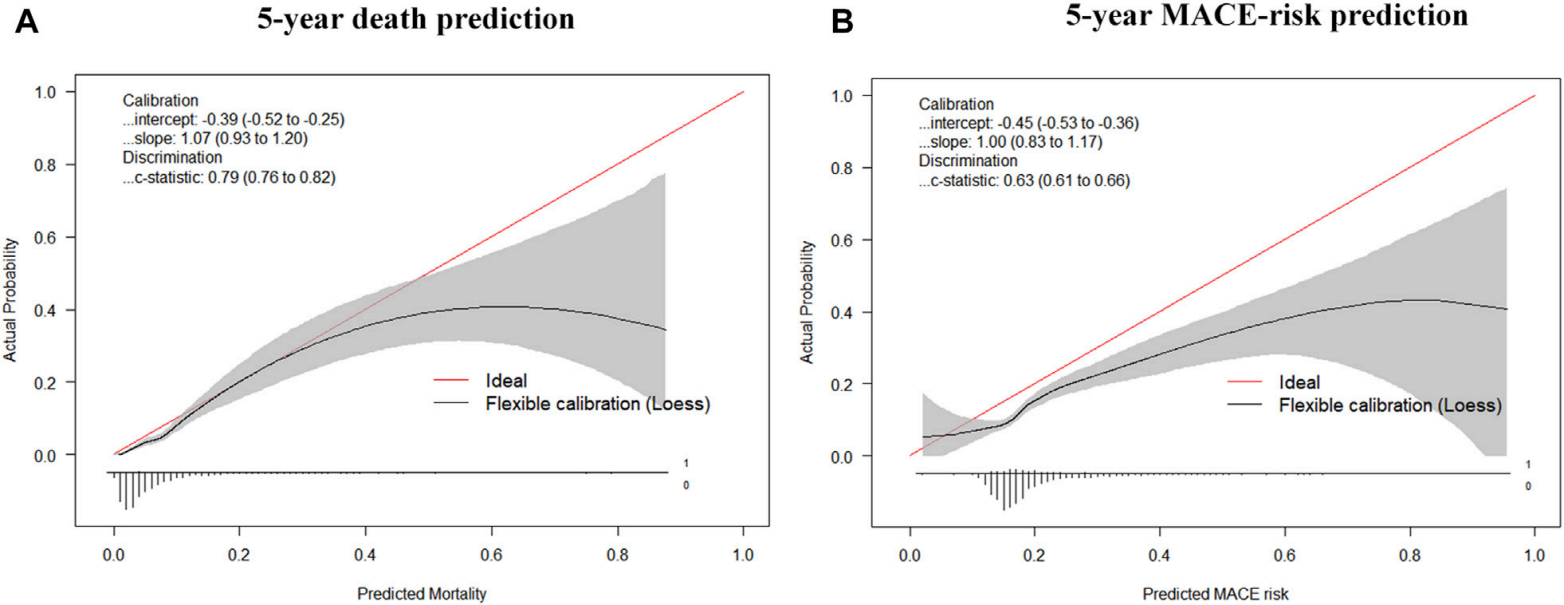
Calibration curves for the nomogram model. **(A)** Calibration curves of the prognostic nomogram for 5-years overall survival; **(B)** Calibration curves of the nomogram predicting risk of MACE.

**FIGURE 7:**
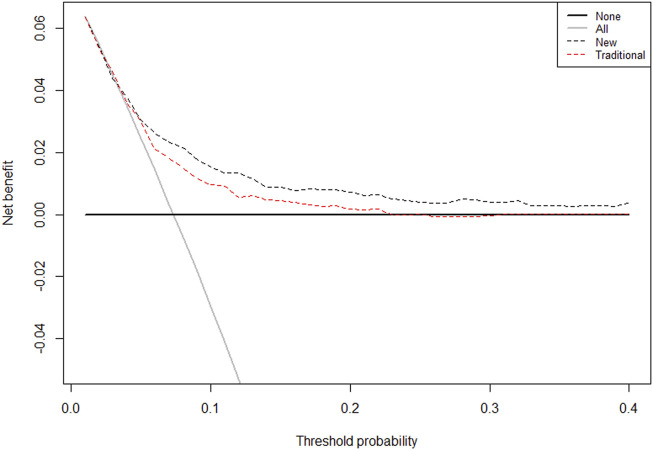
The Decision Curves Analysis (DCA) curve of the prognostic nomogram for predicting probability of overall survival at 5 years. Note: The traditional group-composed by parameters of age, sex, hypertension, diabetes, CHOL, HDLC, BMI, smoking, TRIG; Novel group: age, HF, FT3/FT4, ALB, and proBNP. The horizontal axis represents the threshold value, which is the reference probability of whether a patient receives treatment, and the vertical axis represents the net benefit rate after the advantages minus the disadvantages. Under the same threshold probability, the larger net benefit implies that patients can obtain the maximum benefit using the diagnosis of this model. The closer the curve in the DCA graph is to the top, the higher the value of the model diagnosis will be.

**FIGURE 8:**
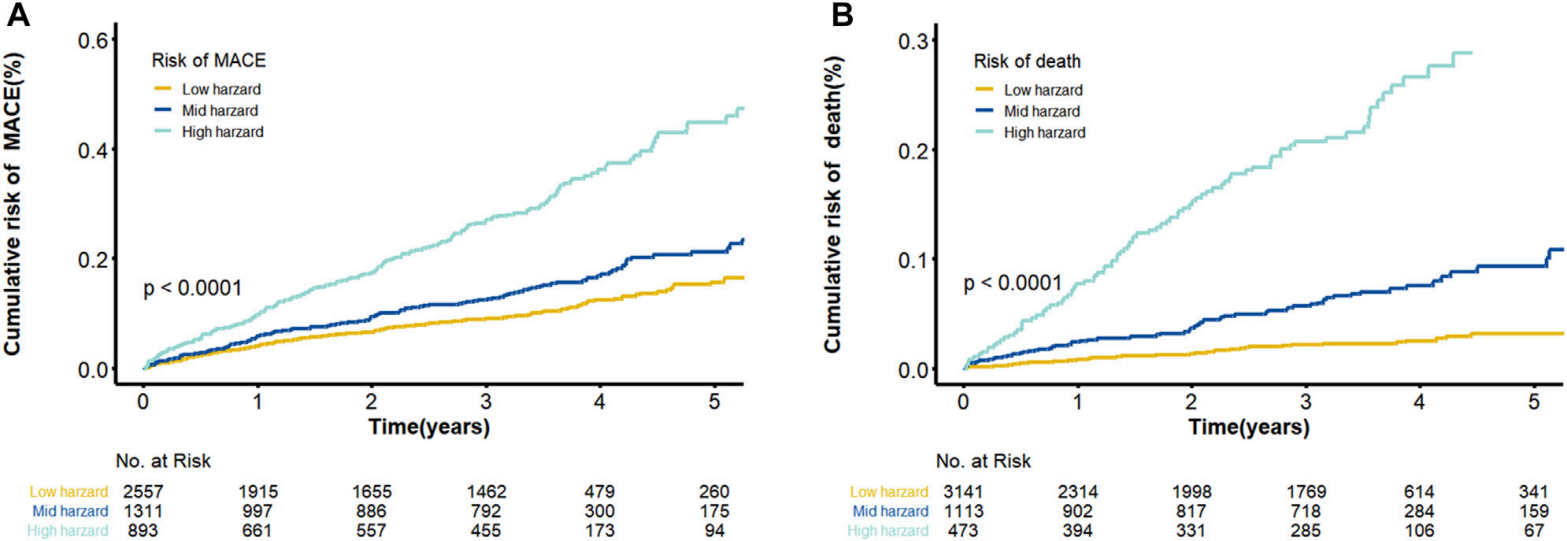
Cumulative Kaplan–Meier curves of the prediction model. **(A)** Cumulative risk of MACE by predicted 5-years ABC-MACE risk group; **(B)** Cumulative probability of mortality by predicted 5-years ABC-death risk group. Note: Survival curves stratified by the score calculated by the nomogram scoring system [low risk, moderate risk and high risk].

The final model was represented by a nomogram, which showed that low FT3/FT4 values; low ALB levels, high FIB levels, and high proBNP levels were associated with low survival rates. The calibration curves of the nomogram for the predicted five-year mortality and MACE risk demonstrated consistency with the prediction and observation of the primary cohort (Figure 6). Decision curve analysis showed that the use of nomogram to predict mortality adds more benefit than either the treat-all-patients scheme or the treat-none scheme (Figure 7). Finally, the prognostic model for death was divided into three groups according to the total score, as follows: low risk (<118), medium risk (118–124) and high risk (≥124). Similarly, the scores of low-, medium-, and high-risk of MACE groups were <113, 113–122, and ≥122, respectively. The cumulative survival curve of risk stratification is shown in Figure 8.

## Discussion

Our study demonstrated that the levels of FT3 and FT3/FT4 were independent predictors of death and MACE risk in patients with CAD on multivariate analyses including conventional CAD risk factors. We also proved that thyroid hormone indicators were significantly related to the emerging biomarkers proBNP, FIB, and ALB, which suggested that thyroid hormone levels may have an impact on adverse outcomes by the key biological pathways, such as heart function and injury, liver function, and coagulation. Finally, according to the predictive characteristics of FT3 and other cardiovascular biomarkers, we developed and validated a new, well-calibrated, biomarker-based risk score to assess the death risk of patients with CAD.

In our study, FT3 and FT3/FT4 are powerful prognostic markers. In recent years, the consequences on the cardiovascular system of milder forms of thyroid dysfunction have been increasingly recognized (Gomberg-Maitland and Frishman, 1998). The further clinical and experimental evidences suggest that a low FT3 level is a strong predictor of a poor prognosis in patients with chronic cardiovascular diseases. However, the relationship between low FT3 and adverse prognosis of CAD patients had a controversy in the last few years (Iervasi et al., 2003; Boelaert and Franklyn, 2005; Coceani et al., 2009; Iervasi, 2009; Ichiki, 2014). In this study, we found that more deaths and MACE occurred in the low FT3 group. Previous studies showed that FT3 promoted the progression of atherosclerosis probably due to the inhibition of lipoprotein enzyme activity and the decreasing clearance rate of total cholesterol (Chapidze et al., 2006; Asvold et al., 2007; Maria Moreno et al., 2019). Although FT3 is associated with lipid levels and linked with the increased mortality of CAD, the mechanism is worthy of further study due to the result of the interaction of many factors (Danese et al., 2000; Duntas, 2002; Erem, 2006).

On the basis of the relationship between FT3 and the prognosis of CAD, we also analyzed the correlation between FT3 and other clinical indicators of CAD patients to further understand whether or not the function was influenced by low FT3 with other common risk factors of CAD. Low T3 syndrome is associated with many traditional risk factors of CAD, such as proBNP, FIB, and ALB (Knezl et al., 2008; Chuang et al., 2014; Brozaitiene et al., 2016). Liang *et al.* reported that BNP was increased six-fold, and its promoter activity increased three to fivefold following T3 treatment (Liang et al., 2003). Afandi *et al.* reported that the decrease in thyroid hormone binding proteins is often a consequence of the acute phase response by impaired synthesis, rapid breakdown, and movement out of the plasma space (Afandi et al., 2000). However, there are no detailed data on thyroid parameters in patients with acute liver failure (ALF) so far. One study showed that more than 50% of ALF patients exhibit abnormal thyroid parameters, and their prognosis was worse than that of normal thyroid patients (Anastasiou et al., 2015). In addition, a study suggested that hyperthyroidism was associated with an increased risk of thrombosis, and patients with hyperthyroidism show higher levels of fibrinogen (Kim et al., 2017). Another study confirmed that VWF and fibrinogen mediate up to 10% of the association between FT4 and cardiovascular disease (Bano et al., 2019). In our study, the level of coagulation parameters we observed changes significantly with the increase of FT4 level, and seems to be less affected by FT3 level. It can be concluded from the existing literature that patients with hypothyroidism are at increased risk of bleeding complications due to impaired coagulation and fibrinolysis (Elbers et al., 2018). Notably, thyroid function with the pathophysiological mechanism of the heart and the prognosis of acute myocardial infarction is still being explored. A study demonstrated a significant relationship between the suppression of thyroid axis function, increased inflammation markers and increased NT-pro-BNP levels in CAD patients undergoing rehabilitation after ACS. They have also demonstrated that measures of FT4 and FT3/FT4 ratio together with NT-pro-BNP can be important prognostic markers of negative long-term outcomes (i.e., mortality) (Brozaitiene et al., 2016). A study aimed at explaining the relationship between T3 and cardiac function found that in the multiple regression analysis, FT3 levels were positively correlated with cardiac ejection fraction, and significantly negatively correlated with NT-proBNP(She et al., 2018). This is consistent with the correlation trend results of FT3 and proBNP obtained in this study. Overall, this study provides further insights into the relationship between thyroid hormones and liver function, thrombosis, and heart function. Whether the effect of thyroid hormones on cardiovascular events and cardiovascular death is mediated by coagulation factors or regulated by heart and liver functions requires further research in the future.

Identifying new biomarkers to effectively improve the prediction for prognosis and guide the individualized treatment of patients with CAD is critical (Malakar et al., 2019). As a quantitative tool for assessing risk and benefit, clinical predictive models provide risk estimates for the presence of the disease (diagnosis) or an event in the future course of disease (prognosis) for individual patients (Steyerberg and Vergouwe, 2014). We developed and validated a well-calibrated new risk score based on biomarkers to assess the risk of death in CAD patients. The novel ABC death score includes age, clinical history of HF, and biomarkers of proBNP, FT3/FT4, and ALB. The developed model including multiple and novel risk factors is useful for estimating prognosis in patients with CHD to inform treatment decisions or for use as a risk stratification tool in future research.

Although this novel ABC–CHD model has few variables, it provides reliable CV death prediction compared with models composed of traditional clinical factors. In our model, the most important prognostic variable is proBNP, which is a well-known marker of stress and dysfunction of cardiomyocytes (Beatty et al., 2015). Recent evidence suggests that proBNP also plays a role in metabolic pathways, including lipolysis and regulation of blood glucose levels, which are important in the pathophysiology of CAD (Zois et al., 2014). A study suggested that proBNP is independent of other prognostic markers, including systolic and diastolic dysfunction, left ventricular mass index, inducible ischemia, exercise capacity, C-reactive protein (CRP), cTnT, and New York Heart Association classification, which can predict cardiovascular morbidity and mortality (Bibbins-Domingo et al., 2007). Increasing concentration of proBNP may also be a signal of vascular dysfunction, in which the natriuretic peptides produce changes in vascular smooth muscle proliferation or contractility, in part via cyclic guanosine monophosphate cascades or nitric oxide synthesis (Ahluwalia et al., 2004).

In summary, these findings suggest that a simple test of biomarkers (i.e., proBNP and ALB) may stratify the risk of prognosis in patients with CAD and provide treatment strategies aimed at reducing future cardiovascular disease morbidity and mortality. The novel model can bring more incremental risk prediction (C index difference: 0.071) than the traditional predictive models of death. Furthermore, considering the powerful performance of the novel ABC-CHD model with a small number of variables provides a tool that may be easier to use in the clinical environment.

This study has several limitations. First, this study was based on a single center. Larger studies enrolling a more diverse population are needed to verify these findings and the external validity of prognostic models. Second, because measurements of FT3, FT4, and TSH were performed only once during initial hospitalization, we were unable to account for possible variations in thyroid function over time. Third, more patients with low FT3 have a history of HF compared with normal patients. Thus, the outcomes of patients may be biased. Further mechanism studies about low FT3 on the risk of death should be considered in the interaction with proBNP, FIB, and ALB. This complex relation merits further well-designed investigations.

## Conclusion

This study demonstrated that low levels of FT3 and FT3/FT4 are independent predictors of death and MACE risk in CAD patients. The associations of thyroid hormone with other functional parameters (i.e. heart function and injury, liver function, and coagulation markers) warrant further study. A novel risk score for the prediction of death including age, HF, and three biomarkers was successfully developed and validated. It can be widely used to complement clinical assessment and guide management based on death and MACE risk stratification in patients with CAD because it is based on a small number of readily available biomarkers and clinical factors.

## Data Availability

The original contributions presented in the study are included in the article/Supplementary Material, further inquiries can be directed to the corresponding author.

## Abbreviations

FT3, free triiodothyronine; FT4, free thyroxine; ALB, albumin; FIB, fibrinogen; proBNP, pro-B-type natriuretic peptide; HR, hazard ratio; 95%CI, 95% confidence interval.
