# Supplementary material for: Free Triiodothyronine Connected With Metabolic Changes in Patients With Coronary Artery Disease by Interacting With Other Functional Indicators
Source: Front Mol Biosci. 2021 Jul 30;8:681955. doi: 10.3389/fmolb.2021.681955 (PMC8362995; doi:10.3389/fmolb.2021.681955)
Supplement: Supplementary file 1 [file Table1.DOCX]

**Table S1. Baseline level difference of related biomarkers between low FT3 and normal FT3**

| **Biomarkers** | **Mean ± SD** | | **P value** |
| --- | --- | --- | --- |
|  | **Low FT3 (N=326)** | **Normal FT3 (N=4778)** |  |
| ProBNP,pg/ml | 3455.39±6904.51 | 806.22±2167.5 | <.0001 |
| ALB,g/L | 32.28±4.5 | 36.98±3.83 | <.0001 |
| HBDH,U/L | 244.26±234.05 | 153.45±127.16 | <.0001 |
| TRIG,mmol/L | 1.61±1.52 | 1.69±1.29 | 0.0007 |
| TP,g/L | 61.05±6.08 | 65.38±5.71 | <.0001 |
| CA,mmol/L | 2.16±0.15 | 2.24±0.11 | <.0001 |
| cTnl(A),ug/L | 6.38±16.18 | 1.51±8.06 | <.0001 |
| DBIL,umol/L | 5±7.09 | 3.73±1.52 | <.0001 |
| HS-CRP,mg/L | 26.92±46.34 | 8.92±20.55 | <.0001 |
| TNT-HS,mg/L | 461.41±999.56 | 200.64±754.62 | <.0001 |
| FIB,g/L | 4.65±1.65 | 3.98±1.16 | <.0001 |
| HCT,% | 0.62±2.94 | 0.45±1.51 | <.0001 |
| HGB,g/L | 120.44±20.72 | 134.78±15.65 | <.0001 |
| LYMPH% | 0.2±0.09 | 0.26±0.08 | <.0001 |
| NEUT%/LYMPH% | 4.68±3.79 | 2.76±2.03 | <.0001 |
| SD = standard deviation; ProBNP = brain-type natriuretic peptide; ALB = albumin; HBDH = hydroxybutyrate dehydrogenase; TRIG = triglyceride; TP = total protein; CA = creatine kinase MB; cTnl = cardiac troponin; DBIL = direct bilirubin; HS-CRP = high-sensitivity C-reactive protein; TNT-HS = high sensitivity troponin T; FIB = fibrinogen; HCT = hematocrit; HGB = hemoglobin; LYMPH%= lymphocyte percentage; NEUT%= neutrophils percentage. | | | |

**Table S2. Univariable Cox proportional hazards analysis of Baseline Characteristics**

| **Characteristics** | **Death** | | **MACE** | |
| --- | --- | --- | --- | --- |
|  | **HR**（95%CI） | **P Value** | **HR**（95%CI） | **P Value** |
| **Demographic data** |  |  |  |  |
| Age | 1.055（1.041-1.069） | 3.33E-15 | 1.014（1.007-1.021） | 0.0002 |
| Sex | 1.098（0.823-1.465） | 0.5248 | 1.218（1.03-1.441） | 0.0212 |
| Smoke | 1.786（1.209-2.639） | 0.0036 | 1.092（0.881-1.353） | 0.4218 |
| BMI | 0.914（0.864-0.966） | 0.0015 | 0.982（0.96-1.004） | 0.1023 |
| **Comorbidities** |  |  |  |  |
| Arrhythmia | 2.11（1.555-2.862） | 1.63E-06 | 1.296（1.056-1.589） | 0.0129 |
| Diabetes | 1.378（1.155-1.643） | 0.0004 | 1.345（1.212-1.492） | 2.54E-08 |
| Heart failure | 4.048（3.071-5.334） | 3.11E-23 | 1.934（1.584-2.36） | 8.60E-11 |
| Hypertension | 1.461（1.133-1.882） | 0.0034 | 1.274（1.108-1.465） | 0.0007 |
| Hyperlipidemia | 0.612（0.388-0.965） | 0.0345 | 1.051（0.855-1.293） | 0.637 |
| **Medication** |  |  |  |  |
| β-blockers | 0.965（0.707-1.318） | 0.8248 | 0.907（0.768-1.072） | 0.2519 |
| ACEIs | 1.088（0.85-1.393） | 0.5019 | 1.025（0.893-1.176） | 0.73 |
| CCBs | 1.552（1.212-1.985） | 0.0005 | 1.432（1.245-1.647） | 5.27E-07 |
| PPIs | 1.291（1.013-1.646） | 0.0392 | 1.207（1.053-1.384） | 0.0068 |
| **biochemical measurements** |  |  |  |  |
| eGFR | 0.979（0.974-0.984） | 4.34E-15 | 0.998（0.997-1） | 0.0487 |
| ALT | 1.001（0.999-1.004） | 0.2539 | 1.001（1-1.003） | 0.1452 |
| AST | 1.002（1-1.003） | 0.0061 | 1.001（1.001-1.002） | 5.15E-05 |
| APOA | 0.366（0.218-0.612） | 0.0001 | 0.532（0.404-0.699） | 6.09E-06 |
| CHOL | 0.875（0.786-0.973） | 0.014 | 1.032（0.979-1.089） | 0.2405 |
| CK | 1（1-1） | 0.0385 | 1（1-1） | 6.00E-04 |
| CKMB | 1.004（1-1.009） | 0.0527 | 1.004（1.002-1.007） | 4.00E-04 |
| GLUC | 1.067（1.039-1.096） | 2.35E-06 | 1.047（1.028-1.066） | 1.01E-06 |
| HDLC | 0.484（0.294-0.797） | 0.0043 | 0.514（0.388-0.68） | 3.17E-06 |
| LDLC | 0.864（0.758-0.985） | 0.0283 | 1.055（0.987-1.128） | 0.1144 |
| LPa | 1.001（1-1.001） | 0.0004 | 1（1-1.001） | 5.30E-06 |
| TRIG | 0.914（0.813-1.028） | 0.1343 | 0.991（0.941-1.042） | 0.7163 |

SD = standard deviation; BMI = body mass index; ALT = alanine aminotransferase; AST = aspartate aminotransferase; CK = creatine kinase; eGFR = estimated glomerular filtration rate; CKMB = creatine kinase MB; CHOL = cholesterol; LDLC = low-density lipoprotein cholesterol; HDLC = high-density lipoprotein cholesterol; TRIG = triglyceride; GLUC = glucose; Lpa = lipoprotein (a); APOA = apolipoprotein a; ACEIs = angiotensin converting enzyme inhibitors; CCBs = calcium channel blockers; PPIs = proton pump inhibitors.

**Table S3. Cox proportional hazards analysis for related biomarkers with FT3**

| **Biomarkers** | **Death** | | **MACE** | |
| --- | --- | --- | --- | --- |
|  | **Univariate analysis** | | | |
|  | **HR（95%CI）** | **P Value** | **HR（95%CI）** | **P Value** |
| ProBNP | 1（1-1） | 1.26E-36 | 1（1-1） | 4.21E-20 |
| ALB | 0.859（0.837-0.881） | 3.98E-32 | 0.933（0.918-0.947） | 6.98E-18 |
| HBDH | 1（1-1） | 2.16E-10 | 1（1-1） | 3.35E-10 |
| TRIG | 0.915（0.814-1.03） | 0.138 | 0.991（0.942-1.04） | 0.722 |
| TP | 0.966（0.946-0.986） | 0.00105 | 0.985（0.974-0.997） | 0.0114 |
| CA | 0.236（0.114-0.49） | 0.000108 | 0.517（0.316-0.845） | 0.00853 |
| cTnI | 0.999（0.981-1.02） | 0.943 | 1（0.993-1.01） | 0.633 |
| DBIL | 1.02（1-1.04） | 0.014 | 1.01（0.988-1.03） | 0.424 |
| HS-CRP | 1.01（1-1.01） | 0.00412 | 1.01（1-1.01） | 9.76E-06 |
| TNT-HS | 1（1-1） | 0.0517 | 1（1-1） | 0.00123 |
| FIB | 1.33（1.23-1.43） | 8.06E-13 | 1.2（1.14-1.26） | 2.69E-13 |
| HCT | 1.06（1.04-1.09） | 0.000000159 | 1.04（1.01-1.06） | 0.00143 |
| HGB | 0.965（0.96-0.971） | 3E-29 | 0.987（0.983-0.991） | 1.93E-10 |
| LYMPH% | 0.00839（0.00352-0.02） | 3.71E-27 | 0.0622（0.0306-0.127） | 1.76E-14 |
| NEUT/LYMPH | 1.09（1.06-1.12） | 5.12E-08 | 1.06（1.04-1.08） | 5.51E-07 |
